# Differential Cardiovascular Outcomes after Dipeptidyl Peptidase-4 Inhibitor, Sulfonylurea, and Pioglitazone Therapy, All in Combination with Metformin, for Type 2 Diabetes: A Population-Based Cohort Study

**DOI:** 10.1371/journal.pone.0124287

**Published:** 2015-05-20

**Authors:** Jong-Mi Seong, Nam-Kyong Choi, Ju-Young Shin, Yoosoo Chang, Ye-Jee Kim, Joongyub Lee, Ju-Young Kim, Byung-Joo Park

**Affiliations:** 1 Office of Drug Safety Information II, Korea Institute of Drug Safety & Risk Management, Seoul, Republic of Korea; 2 Department of Preventive Medicine, Seoul National University College of Medicine, Seoul, Republic of Korea; 3 Division of Clinical Epidemiology, Medical Research Collaborating Center, Seoul National University College of Medicine/Seoul National University Hospital, Seoul, Republic of Korea; 4 Medical Research Center, Seoul National University, Seoul, Republic of Korea; 5 Office of Drug Utilization Review, Korea Institute of Drug Safety & Risk Management, Seoul, Republic of Korea; 6 Department of Occupational and Environmental Medicine, Kangbuk Samsung Hospital, Sungkyunkwan University, School of Medicine, Seoul, Republic of Korea; 7 Office of Drug Safety Information I, Korea Institute of Drug Safety & Risk Management, Seoul, Republic of Korea; 8 Department of Family Medicine, Seoul National University Bundang Hospital, Seoul, Republic of Korea; National University of Singapore, SINGAPORE

## Abstract

**Background/Objectives:**

Data on the comparative effectiveness of oral antidiabetics on cardiovascular outcomes in a clinical practice setting are limited. This study sought to determine whether a differential risk of cardiovascular disease (CVD) exists for the combination of a dipeptidyl peptidase-4 (DPP-4) inhibitor plus metformin versus a sulfonylurea derivative plus metformin or pioglitazone plus metformin.

**Methods:**

We conducted a cohort study of 349,476 patients who received treatment with a DPP-4 inhibitor, sulfonylurea, or pioglitazone plus metformin for type 2 diabetes using the Korean national health insurance claims database. The incidence of total CVD and individual outcomes of myocardial infarction (MI), heart failure (HF), and ischemic stroke (IS) were assessed using the hazard ratios (HRs) estimated from a Cox proportional-hazards model weighted for a propensity score.

**Results:**

During follow-up, 3,881 patients developed a CVD, including 428 MIs, 212 HFs, and 1,487 ISs. The adjusted HR with 95% confidence interval (CI) for a sulfonylurea derivative plus metformin compared with a DPP-4 inhibitor plus metformin was 1.20 (1.09-1.32) for total CVD; 1.14 (1.04-1.91) for MI; 1.07 (0.71-1.62) for HF; and 1.51 (1.28-1.79) for IS. The HRs with 95% CI for total CVD, MI, HF, and IS for pioglitazone plus metformin were 0.89 (0.81-0.99), 1.05 (0.76-1.46), 4.81 (3.53-6.56), and 0.81 (0.67-0.99), respectively.

**Conclusions:**

Compared with a DPP-4 inhibitor plus metformin, treatment with a sulfonylurea drug plus metformin was associated with increased risks of total CVD, MI, and IS, whereas the use of pioglitazone plus metformin was associated with decreased total CVD and IS risks.

## Introduction

The incidence of type 2 diabetes is increasing worldwide, which imposes a high burden of morbidity and mortality, mainly due to cardiovascular disease (CVD). Patients with diabetes have an increased risk of developing CVD, which is also the leading cause of mortality in patients with diabetes [1–3]. Therefore, it is important that the effects of diabetes therapies on reducing the CVD risk be characterized.

Dipeptidyl peptidase-4 (DPP-4) inhibitors are a class of agents that were recently approved for the treatment of type 2 diabetes. These agents extend the action of insulin while also suppressing the release of glucagon via increasing the bioactive form of the incretins, glucagon-like peptide-1 and glucose-dependent insulinotropic polypeptide, thereby improving glucose homeostasis [4–5]. DPP‑4 inhibitors provide comparable efficacy to other oral antidiabetics (OAs) [6] and carry lower risks of hypoglycemia and weight gain [6–7]. Meta-analyses of randomized controlled trials (RCTs) have indicated that DPP-4 inhibitors might reduce the risk of major cardiovascular events (CVEs) compared with a placebo or other OAs [8–10]. However, among the RCTs included in these meta-analyses, CVEs were reported only as adverse events and were not pre-specified as primary outcomes. Recently, post-marketing trials of DPP-4 inhibitors have shown that these drugs neither reduced nor increased the risk of major CVEs compared with placebo [11–12]. However, the results from patients enrolled in RCTs may not be generalized to the general population. Thus, cardiovascular outcome data from patients in real-world clinical settings are needed.

We performed this population-based cohort study to evaluate the differential risk of CVDs between the use of DPP-4 inhibitors and other OAs using the national health insurance claims database. Given the common and increasing use of combined OAs [13], this study focused on the administration of a DPP-4 inhibitor in combination with metformin, and this therapy was compared to treatment with a sulfonylurea derivative and metformin or pioglitazone and metformin, which have been the most commonly prescribed therapies in dual-therapy users. Study drugs were approved for the treatment of type 2 diabetes as monotherapy or combination therapy, whereas prescribing patterns of these drugs reflect the reimbursement criteria; metformin monotherapy is considered as the first-line drug for type 2 diabetes and covered by Korean health insurance program; a DPP-4 inhibitor, sulfonylurea drug or pioglitazone is generally used as combination therapy with metformin or other OAs.

## Materials and Methods

### Study Design, Setting, and Population

The source population for this retrospective cohort study was derived from the Health Insurance Review & Assessment Service (HIRA) database, which represents the national health insurance claims database covering approximately 50 million Koreans [14–15]. We obtained claims data that had been submitted for patients by healthcare providers between January 1, 2006 and December 31, 2010; these data contained anonymized identifiers given by HIRA to protect the patient’s privacy, according to the Act on the Protection of Personal Information Maintained by Public Agencies. The database contains longitudinal patient data including patient demographics, diagnoses (International Classification of Disease, Tenth Revision [ICD-10]), procedures, prescription drugs (brand name, generic name, prescription date, days of supply, dose, and route of administration), and type of medical utilization (outpatient, inpatient, or emergency department).

**Fig 1:**
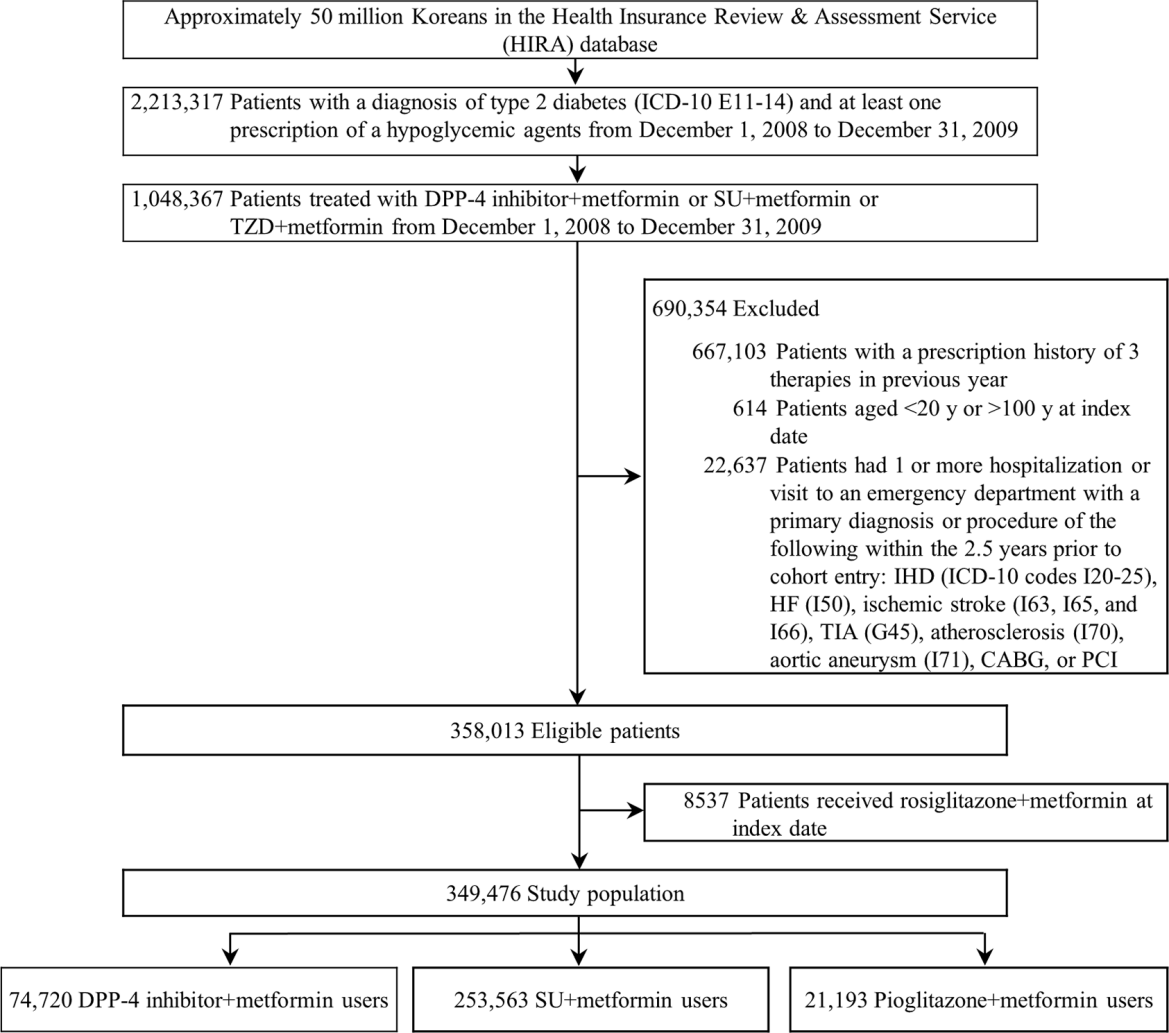
Selection of the study population. ICD-10 = the International Classification of Disease, Tenth Revision; SU = sulfonylurea; TZD = thiazolidinedione; DPP-4 = dipeptidyl peptidase-4; IHD = ischemic heart disease; HF = heart failure; TIA = transient ischemic attack; CABG = coronary artery bypass grafts; PCI = percutaneous coronary intervention.

The study population consisted of type 2 diabetes patients (ICD-10 codes E11-14) who were newly treated with the study therapies between December 1, 2008 (the date that the DPP-4 inhibitors were introduced into the national insurance coverage) and December 31, 2009. These subjects ranged in age from 20 to 99 years at cohort entry (Fig 1). The study therapies were defined as follows: a DPP-4 inhibitor (sitagliptin or vildagliptin) plus metformin, a sulfonylurea drug (glibenclamide, glipizide, gliquidone, gliclazide, or glimepiride) plus metformin, or pioglitazone plus metformin. We identified all prescriptions for the study therapies. For all eligible patients, the index date was defined as the date when any of these 3 study therapies was first prescribed. To include only patients that were newly treated with the study therapies, patients who had received any of these 3 therapies in the year before the index date were excluded. To identify incident CVD, patients with a history of CVD within the 2.5 years prior to cohort entry were also excluded.

The study patients were classified into the following three study groups: DPP-4 inhibitor (DPP-4 inhibitor+metformin), sulfonylurea (SU+metformin), and pioglitazone (pioglitazone+metformin). Patients receiving rosiglitazone plus metformin were excluded because the use of rosiglitazone has been significantly limited in Korea.

### Exposure Assessment

A patient was considered continuously exposed to a study therapy if he or she filled a prescription for that therapy within the end date of the previous prescription plus 1.5 times the prescription days’ supply [16]. If a patient did not refill the prescription within this time (therapy stop) or the patient filled a prescription for a different therapy or made an addition and/or subtraction of any hypoglycemic agent to an existing therapy (therapy switch), he or she was considered to have discontinued the study therapy. The therapy discontinuation date for therapy stop was set as 1.5 times the prescription days’ supply after the end of the days’ supply from the most recent prescription of a study therapy. For a therapy switch, the therapy discontinuation date was the date a different therapy was prescribed.

### Study Outcomes

The primary outcome of interest was any CVD, defined as hospitalization or a visit to an emergency department with a primary diagnosis of ischemic heart disease (ICD-10 codes I20-25), heart failure (I50; HF), ischemic stroke (I63, I65, and I66; IS), transient ischemic attack (G45), atherosclerosis (I70), or aortic aneurysm (I71) or for treatment with coronary artery bypass grafts or percutaneous coronary intervention. The secondary outcomes consisted of individual diseases including myocardial infarction (MI), HF, and IS. The secondary outcomes were defined as hospitalization or a visit to an emergency department with a primary diagnosis of each outcome (ICD-10 codes for MI: I21-23; for HF: I50; for IS: I63, I65, and I66). The outcome date was the earliest date a patient encountered a given outcome.

### Covariates

The study covariates included age at index date, gender, duration of diabetes, presence of comorbidities, and use of the medications specified below. The duration of diabetes was assessed based on the date of the first medical claim with a diagnosis code for diabetes (ICD-10 codes E11-14) during the 2.5 years prior to the index date. Comorbidities were determined by ICD-10 codes for the following conditions in the 1 year prior to the index date: microvascular complications of diabetes (retinopathy, neuropathy, or nephropathy), peripheral vascular disease, hypertension, and dyslipidemia (S1 Table). Obesity was assessed based on any medical claim with diagnosis of obesity (ICD-10 code E66). The Charlson comorbidity score was calculated in the year before the index date [17]. We assessed whether a patient had a diagnosis of family history of diabetes mellitus (Z83.3) from January 1, 2006 to the end date of cohort entry. We also assessed whether a patient was hospitalized with a primary diagnosis of diabetes and the total number of hypoglycemic agent classes used in the year prior to the index date. Finally, we assessed the use of the following medications in the year prior to the index date: metformin, sulfonylureas, thiazolidinediones, α-glucosidase inhibitors, meglitinides, DPP-4 inhibitors, insulin, angiotensin converting enzyme inhibitors, angiotensin receptor blockers (ARBs), β-blockers, calcium channel blockers, thiazide diuretics, other diuretics, nitrates, digoxin, aspirin, other antiplatelet drugs, warfarin, statins, and nonsteroidal anti-inflammatory drugs.

### Follow-up and Statistical Analysis

Follow-up for each patient started on the index date and ended at the earliest occurrence of a study outcome, therapy discontinuation (therapy stop or switch), death (in-hospital death), or the end of the study period (December 31, 2010).

Baseline characteristics of each study group were compared using standardized mean differences (SMD), calculated as the difference in means or proportions of a variable divided by a pooled estimate of the standard deviation of the variable. A value of 0.1 SMD or less indicates a negligible difference in means between groups [18].

Incidence rates and 95% confidence intervals (CI) of a study outcome for each of the study groups were calculated. We compared the DPP-4 inhibitor group with both the sulfonylurea and pioglitazone groups. Hazard ratios (HRs) with 95% CIs were calculated using Cox proportional hazards models. Propensity score analyses were used to balance confounders between the group receiving a DPP-4 inhibitor plus metformin and each of the 2 groups receiving a sulfonylurea drug plus metformin and pioglitazone plus metformin. The propensity scores were estimated for each patient by fitting a logistic regression model to predict a DPP-4 inhibitor plus metformin versus a sulfonylurea drug or pioglitazone plus metformin initiation, as a function of the baseline covariates. Among all covariates (Table 1), variables associated with a greater than 20% increase or reduction in each outcome rate were included in the propensity score [19–20]. We then weighted a Cox model based on propensity score values; in these analyses, we used standardized morbidity ratio (SMR) weights [21–22]. The SMR weighting method estimates the treatment effect in a population whose distribution of risk factors is equal to that found in the treated study patients. These SMR weighted analyses use as weights the value 1 for the treated (that is, users of a DPP-4 inhibitor plus metformin) and the propensity odds for the untreated (users of a sulfonylurea or pioglitazone plus metformin). To estimate the gender-specific HRs and 95% CIs, we stratified the analysis by gender (male and female). All statistical analyses were performed using SAS software Version 9.3 (SAS Institute Inc., Cary, NC, USA).

**Table 1:** Baseline characteristics of the study population.

Characteristics		DPP-4 inhibitor+metformin(N = 74,720)		SU+metformin(N = 253,563)		Pioglitazone+metformin(N = 21,193)		SMD_SU_	SMD_PIO_
Gender, n (%)	Male	39,865	(53.4)	139,497	(55.0)	11,805	(55.7)	0.03	0.05
	Female	34,855	(46.6)	114,066	(45.0)	9,388	(44.3)	0.03	0.05
Age, mean (SD), y		57.0	(12.0)	58.7	(12.5)	57.3	(12.1)	0.14	0.02
Age group, n (%)	20–44	11,405	(15.3)	34,136	(13.5)	3,142	(14.8)	0.05	0.01
	45–64	41,846	(56.0)	129,886	(51.2)	11,644	(54.9)	0.10	0.02
	65+	21,469	(28.7)	89,541	(35.3)	6,407	(30.2)	0.14	0.03
Duration of diabetes, y, n (%)	< 1	18,124	(24.3)	73,438	(29.0)	6,421	(30.3)	0.11	0.14
	1–2.5	11,211	(15.0)	38,576	(15.2)	3,729	(17.6)	0.01	0.07
	> 2.5	45,385	(60.7)	141,549	(55.8)	11,043	(52.1)	0.10	0.17
Microvascular complications in previous year, n (%)	Retinopathy	3,188	(4.3)	7,963	(3.1)	654	(3.1)	0.06	0.06
	Neuropathy	7,078	(9.5)	18,226	(7.2)	1,802	(8.5)	0.08	0.03
	Nephropathy	1,965	(2.6)	3,283	(1.3)	423	(2.0)	0.10	0.04
Other comorbidities in previous year, n (%)	Peripheral vascular disease	6,901	(9.2)	17,718	(7.0)	1,795	(8.5)	0.08	0.03
	Hypertension	17,973	(24.1)	68,832	(27.1)	5,929	(28.0)	0.07	0.09
	Dyslipidemia	1,352	(1.8)	3,493	(1.4)	448	(2.1)	0.03	0.02
	Obesity	545	(0.7)	1,261	(0.5)	170	(0.8)	0.03	0.01
Charlson score, mean (SD)		1.2	(1.3)	1.0	(1.2)	1.0	(1.2)	0.16	0.16
Family history of diabetes mellitus		153	(0.2)	410	(0.2)	48	(0.2)	0.01	0.00
DM-related hospitalization, n (%)		2,690	(3.6)	9,259	(3.7)	649	(3.1)	0.00	0.03
Total number of hypoglycemic agents used, mean (SD)		1.8	(1.3)	1.5	(1.3)	1.6	(1.3)	0.38	0.15
Use of hypoglycemic agents in previous year, n (%)	Metformin	46,196	(61.8)	99,973	(39.4)	11,071	(52.2)	0.45	0.19
	Sulfonylureas	35,002	(46.8)	154,510	(60.9)	8,966	(42.3)	0.28	0.09
	Thiazolidinediones	9,772	(13.1)	19,108	(7.5)	6,229	(29.4)	0.18	0.40
	α-glucosidase inhibitors	20,507	(27.4)	53,187	(21.0)	3,990	(18.8)	0.15	0.20
	Meglitinides	6,624	(8.9)	12,263	(4.8)	1,193	(5.6)	0.16	0.12
	DPP-4 inhibitor	4,923	(6.6)	965	(0.4)	64	(0.3)	0.34	0.34
	Insulin	10,551	(14.1)	34,907	(13.8)	2,336	(11.0)	0.01	0.09
History of drug use in previous year, n (%)	Angiotensin converting enzyme inhibitors	6,505	(8.7)	23,878	(9.4)	1,981	(9.3)	0.02	0.02
	Angiotensin receptor antagonists	27,281	(36.5)	74,678	(29.5)	7,274	(34.3)	0.15	0.05
	β adrenergic antagonists	12,535	(16.8)	44,869	(17.7)	3,713	(17.5)	0.02	0.02
	Calcium channel blockers	25,374	(34.0)	92,345	(36.4)	7,408	(35.0)	0.05	0.02
	Thiazide diuretics	18,279	(24.5)	62,549	(24.7)	5,339	(25.2)	0.00	0.02
	Other diuretics	7,230	(9.7)	26,047	(10.3)	2,068	(9.8)	0.02	0.00
	Nitrates	1,570	(2.1)	4,862	(1.9)	301	(1.4)	0.01	0.05
	Digoxin	1,201	(1.6)	4,450	(1.8)	264	(1.2)	0.01	0.03
	Aspirin	24,502	(32.8)	74,212	(29.3)	6,233	(29.4)	0.08	0.07
	Other antiplatelet drugs	15,770	(21.1)	42,651	(16.8)	4,335	(20.5)	0.11	0.02
	Warfarin	585	(0.8)	1,600	(0.6)	88	(0.4)	0.02	0.05
	Statins	28,645	(38.3)	67,063	(26.4)	7,242	(34.2)	0.25	0.09
	Nonsteroidal anti-inflammatory drugs	57,055	(76.4)	191,675	(75.6)	16,466	(77.7)	0.02	0.03

DPP-4 = dipeptidyl peptidase-4; SU = sulfonylurea; SMD_SU_ = standardized mean differences between DPP-4 inhibitor and sulfonylurea therapy groups; SMD_PIO_ = standardized mean differences between DPP-4 inhibitor and pioglitazone therapy groups; SD = standard deviation; DM = diabetes mellitus.

### Ethical Approval

This study protocol was approved by the Institutional Review Board of the Seoul National University College of Medicine/Seoul National University Hospital (IRB No. 1206-056-414). Informed consent was waived by the IRB.

## Results

### Characteristics of Study Population

A total of 349,476 patients were included in the cohort, including 74,720 patients treated with a DPP-4 inhibitor plus metformin, 253,563 treated with a sulfonylurea derivative plus metformin, and 21,193 treated with pioglitazone plus metformin (Fig 1). Patients in the DPP-4 inhibitor group demonstrated a longer duration of diabetes, a higher Charlson comorbidity score, and a larger number of hypoglycemic agents used compared with both the sulfonylurea and pioglitazone groups. Patients using a DPP-4 inhibitor plus metformin were also younger and more likely to have prescriptions for ARBs, other antiplatelet drugs, and statins than the sulfonylurea plus metformin users (Table 1).

### Incidence Rate of Study Outcomes

Study patients contributed between 210,878 and 211,959 person-years of follow-up, depending which the study outcome was analyzed. During follow-up, 3,881 patients developed a CVD, including 428 MIs, 212 HFs, and 1,487 ischemic strokes. Among the patients treated with a DPP-4 inhibitor plus metformin, the crude incidence per 1,000 person-years was 15.75 for total CVD, 1.45 for MI, 0.88 for HF, and 4.65 for ischemic stroke. The crude incidence per 1,000 person-years was 19.88 for total CVD, 2.30 for MI, 1.05 for HF, and 8.24 for IS among patients using a sulfonylurea drug plus metformin, and these values were 13.44 for total CVD, 1.31 for MI, 1.10 for HF, and 3.88 for IS among pioglitazone plus metformin users (Table 2).

**Table 2:** Incidence rates and relative risks of total cardiovascular disease, myocardial infarction, heart failure, and ischemic stroke in patients treated with a DPP-4 inhibitor plus metformin vs. sulfonylurea derivative plus metformin and pioglitazone plus metformin.

Study Outcomes	Study Therapies	Person-Years	No. of Events	Incidence Rate per 1000 Person-Years	Unadjusted HR (95% CI)	Propensity Score-Adjusted HR (95% CI) ^a^ ^-^ ^h^
Total CVD	DPP-4 inhibitor+metformin	55,551	875	15.75	1.00	1.00
	SU+metformin	142,461	2,833	19.88	1.23 (1.14–1.32)	1.20 (1.09–1.32)
	Pioglitazone+metformin	12,866	173	13.44	0.84 (0.71–0.99)	0.89 (0.81–0.99)
MI	DPP-4 inhibitor+metformin	55,813	81	1.45	1.00	1.00
	SU+metformin	143,176	330	2.30	1.56 (1.23–1.99)	1.41 (1.04–1.91)
	Pioglitazone+metformin	12,913	17	1.31	0.91 (0.54–1.53)	1.05 (0.76–1.46)
HF	DPP-4 inhibitor+metformin	55,818	49	0.88	1.00	1.00
	SU+metformin	143,230	150	1.05	1.14 (0.83–1.58)	1.07 (0.71–1.62)
	Pioglitazone+metformin	12,911	13	1.01	1.10 (0.60–2.03)	4.81 (3.53–6.56)
Ischemic Stroke	DPP-4 inhibitor+metformin	55,756	259	4.65	1.00	1.00
	SU+metformin	142,940	1,178	8.24	1.73 (1.51–1.98)	1.51 (1.28–1.79)
	Pioglitazone+metformin	12,903	50	3.88	0.82 (0.60–1.11)	0.81 (0.67–0.99)

DPP-4 = dipeptidyl peptidase-4; SU = sulfonylurea; HR = hazard ratio; CI = confidence interval; CVD = cardiovascular disease; MI = myocardial infarction; HF = heart failure.

^a^ The propensity of receiving dipeptidyl peptidase-4 (DPP-4) inhibitor plus metformin was estimated using a multivariable logistic regression model that included baseline age, age^2^, gender, retinopathy, family history of diabetes mellitus, diabetes-related hospitalization, α-glucosidase inhibitors, insulin, angiotensin converting enzyme inhibitors (ACEIs), angiotensin receptor antagonists (ARBs), β adrenergic antagonists (BBs), calcium channel blockers (CCBs), thiazide diuretics, other diuretics, nitrates, digoxin, aspirin, other antiplatelet drugs, warfarin, and statins and was compared to sulfonylurea therapy in the total cardiovascular disease outcome analysis (C-statistic = 0.613).

^b^ The propensity of receiving a DPP-4 inhibitor plus metformin was estimated using a multivariable logistic regression model that included baseline age, age^2^, gender, duration of diabetes, retinopathy, nephropathy, hypertension, dyslipidemia, obesity, family history of diabetes mellitus, diabetes-related hospitalization, sulfonylureas (SUs), α-glucosidase inhibitors, DPP-4 inhibitor, meglitinides, insulin, ACEIs, BBs, CCBs, nitrates, digoxin, aspirin, other antiplatelet drugs, and warfarin and was compared to SU plus metformin in the myocardial infarction (MI) outcome analysis (C-statistic = 0.673).

^c^ The propensity of receiving a DPP-4 inhibitor plus metformin was estimated using a multivariable logistic regression model that included baseline age, age^2^, gender, duration of diabetes, neuropathy, nephropathy, dyslipidemia, Charlson score, diabetes-related hospitalization, total number of hypoglycemic agents used, metformin, SUs, thiazolidinediones (TZDs), α-glucosidase inhibitors, meglitinides, insulin, ACEIs, ARBs, BBs, other diuretics, nitrates, digoxin, aspirin, other antiplatelet drugs, warfarin, and statins and was compared to SU plus metformin in the heart failure (HF) outcome analysis (C-statistic = 0.720).

^d^ The propensity of receiving a DPP-4 inhibitor plus metformin was estimated using a multivariable logistic regression model that included baseline age, age^2^, gender, duration of diabetes, retinopathy, obesity, diabetes-related hospitalization, DPP-4 inhibitor, insulin, ACEI, BB, CCB, thiazide diuretics, other diuretics, nitrates, digoxin, other antiplatelet drugs, and warfarin and was compared to SU plus metformin in the ischemic stroke analysis (C-statistic = 0.601).

^e^ The propensity of receiving a DPP-4 inhibitor plus metformin was estimated using a multivariable logistic regression model that included baseline age, age^2^, gender, retinopathy, nephropathy, hypertension, α-glucosidase inhibitors, TZDs, insulin, ACEIs, ARBs, BBs, CCBs, thiazide diuretics, other diuretics, nitrates, digoxin, aspirin, other antiplatelet drugs, warfarin, statins, and nonsteroidal anti-inflammatory drugs and was compared to pioglitazone plus metformin in the total cardiovascular disease outcome analysis (C-statistic = 0.646).

^f^ The propensity of receiving a DPP-4 inhibitor plus metformin was estimated using a multivariable logistic regression model that included baseline age, age^2^, gender, duration of diabetes, retinopathy, neuropathy, hypertension, obesity, diabetes-related hospitalization, metformin, TZDs, α-glucosidase inhibitors, meglitinides, DPP-4 inhibitor, insulin, ACEIs, BBs, CCBs, other diuretics, nitrates, digoxin, other antiplatelet drugs, and warfarin and was compared to pioglitazone plus metformin in the MI outcome analysis (C-statistic = 0.672).

^g^ The propensity of receiving a DPP-4 inhibitor plus metformin was estimated using a multivariable logistic regression model that included baseline age, age^2^, gender, duration of diabetes, retinopathy, neuropathy, nephropathy, peripheral vascular disease, hypertension, dyslipidemia, diabetes-related hospitalization, total number of hypoglycemic agents used, Charlson score, metformin, SUs, meglitinides, DPP-4 inhibitor, insulin, ACEIs, ARBs, BBs, CCBs, other diuretics, nitrates, digoxin, other antiplatelet drugs, warfarin, and statins and was compared to pioglitazone plus metformin in the HF outcome analysis (C-statistic = 0.640).

^h^ The propensity of receiving a DPP-4 inhibitor plus metformin was estimated using a multivariable logistic regression model that included baseline age, age^2^, gender, retinopathy, nephropathy, hypertension, dyslipidemia, diabetes-related hospitalization, metformin, SUs, α-glucosidase inhibitors, DPP-4 inhibitor, insulin, ACEIs, ARBs, BBs, CCBs, thiazide diuretics, other diuretics, digoxin, aspirin, other antiplatelet drugs, and warfarin and was compared to pioglitazone plus metformin in the ischemic stroke outcome analysis (C-statistic = 0.606).

### Hazard Ratios in the Full Cohort and in the Gender-specific group

In the models weighted for a propensity score, the HRs of total CVD, MI, and IS for a sulfonylurea derivative plus metformin compared with a DPP-4 inhibitor plus metformin were 1.20 (95% CI, 1.09–1.32), 1.41 (95% CI, 1.04–1.91), and 1.51 (95% CI, 1.28–1.79), respectively. The HRs of total CVD, HF, and IS for pioglitazone plus metformin compared with a DPP-4 inhibitor plus metformin were 0.89 (95% CI, 0.81–0.99), 4.81 (95% CI, 3.53–6.56), and 0.81 (95% CI, 0.67–0.99), respectively (Table 2). Gender-specific HRs are shown in Table 3. The HR of MI for a sulfonylurea drug plus metformin compared with a DPP-4 inhibitor plus metformin was 1.10 (95% CI, 0.75–1.59) among males and 2.42 (95% CI, 1.38–4.25) among females. The HR for total CVD for pioglitazone plus metformin compared with a DPP-4 inhibitor plus metformin was 0.75 (95% CI, 0.65–0.86) among males and 1.09 (95% CI, 0.94–1.26) among females. The corresponding HR for HF was 1.08 (95% CI, 0.52–2.24) among males and 6.53 (95% CI, 4.54–9.40) among females, and the corresponding HR for IS was 0.66 (95% CI, 0.49–0.89) among males and 0.96 (95% CI, 0.74–1.25) among females.

**Table 3:** Subgroup analyses for total cardiovascular disease, myocardial infarction, heart failure, and ischemic stroke in patients treated with a DPP-4 inhibitor plus metformin vs. sulfonylurea drug plus metformin and pioglitazone plus metformin.

Subgroup-Study outcome	Study Therapies	Person-Years	No. of Events	Incidence Rate per 1000 Person-Years	Unadjusted HR (95% CI)	Propensity Score-Adjusted HR (95% CI) ^a^ ^-^ ^h^
Male-Total CVD	DPP-4 inhibitor+metformin	29,600	498	16.82	1.00	1.00
	SU+metformin	78,782	1,564	19.85	1.15 (1.04–1.27)	1.14 (1.00–1.29)
	Pioglitazone+metformin	7,304	91	12.46	0.73 (0.58–0.91)	0.75 (0.65–0.86)
Male-MI	DPP-4 inhibitor+metformin	29,750	62	2.08	1.00	1.00
	SU+metformin	79,160	218	2.75	1.31 (0.99–1.74)	1.10 (0.75–1.59)
	Pioglitazone+metformin	7,326	12	1.64	0.79 (0.42–1.40)	0.88 (0.60–1.29)
Male-HF	DPP-4 inhibitor+metformin	29,761	15	0.50	1.00	1.00
	SU+metformin	79,202	57	0.72	1.37 (0.78–2.43)	1.07 (0.50–2.28)
	Pioglitazone+metformin	7,329	4	0.55	1.05 (0.35–3.17)	1.08 (0.52–2.24)
Male-Ischemic Stroke	DPP-4 inhibitor+metformin	29,729	124	4.17	1.00	1.00
	SU+metformin	79,043	644	8.15	1.92 (1.59–2.33)	1.76 (1.39–2.22)
	Pioglitazone+metformin	7,323	22	3.00	0.71 (0.45–1.12)	0.66 (0.49–0.89)
Female-Total CVD	DPP-4 inhibitor+metformin	25,950	377	14.53	1.00	1.00
	SU+metformin	63,680	1,269	19.93	1.33 (1.18–1.49)	1.28 (1.11–1.48)
	Pioglitazone+metformin	5,562	82	14.74	0.99 (0.78–1.26)	1.09 (0.94–1.26)
Female-MI	DPP-4 inhibitor+metformin	26,063	19	0.73	1.00	1.00
	SU+metformin	64,017	112	1.75	2.33 (1.43–3.80)	2.42 (1.38–4.25)
	Pioglitazone+metformin	5,587	5	0.89	1.23 (0.46–3.30)	1.58 (0.86–2.91)
Female-HF	DPP-4 inhibitor+metformin	26,056	34	1.30	1.00	1.00
	SU+metformin	64,028	93	1.45	1.06 (1.72–1.57)	1.07 (0.65–1.76)
	Pioglitazone+metformin	5,583	9	1.61	1.17 (0.56–2.44)	6.53 (4.54–9.40)
Female-Ischemic Stroke	DPP-4 inhibitor+metformin	26,026	135	5.19	1.00	1.00
	SU+metformin	63,897	534	8.36	1.55 (1.29–1.88)	1.29 (1.01–1.64)
	Pioglitazone+metformin	5,580	28	5.02	0.94 (0.62–1.41)	0.96 (0.74–1.24)

DPP-4 = dipeptidyl peptidase-4; SU = sulfonylurea; HR = hazard ratio; CI = confidence interval; CVD = cardiovascular disease; MI = myocardial infarction; HF = heart failure.

^a^ The propensity of receiving a dipeptidyl peptidase-4 (DPP-4) inhibitor plus metformin was estimated using a multivariable logistic regression model that included baseline age, age^2^, gender, retinopathy, family history of diabetes mellitus, diabetes-related hospitalization, α-glucosidase inhibitors, insulin, angiotensin converting enzyme inhibitors (ACEIs), angiotensin receptor antagonists (ARBs), β adrenergic antagonists (BBs), calcium channel blockers (CCBs), thiazide diuretics, other diuretics, nitrates, digoxin, aspirin, other antiplatelet drugs, warfarin, and statins and was compared to sulfonylurea therapy in the total cardiovascular disease outcome analysis (C-statistic = 0.613).

^b^ The propensity of receiving a DPP-4 inhibitor plus metformin was estimated using a multivariable logistic regression model that included baseline age, age^2^, gender, duration of diabetes, retinopathy, nephropathy, hypertension, dyslipidemia, obesity, family history of diabetes mellitus, diabetes-related hospitalization, sulfonylureas (SUs), α-glucosidase inhibitors, DPP-4 inhibitor, meglitinides, insulin, ACEIs, BBs, CCBs, nitrates, digoxin, aspirin, other antiplatelet drugs, and warfarin and was compared to SU plus metformin in the myocardial infarction (MI) outcome analysis (C-statistic = 0.673).

^c^ The propensity of receiving a DPP-4 inhibitor plus metformin was estimated using a multivariable logistic regression model that included baseline age, age^2^, gender, duration of diabetes, neuropathy, nephropathy, dyslipidemia, Charlson score, diabetes-related hospitalization, total number of hypoglycemic agents used, metformin, SUs, thiazolidinediones (TZDs), α-glucosidase inhibitors, meglitinides, insulin, ACEIs, ARBs, BBs, other diuretics, nitrates, digoxin, aspirin, other antiplatelet drugs, warfarin, and statins and was compared to SU plus metformin in the heart failure (HF) outcome analysis (C-statistic = 0.720).

^d^ The propensity of receiving a DPP-4 inhibitor plus metformin was estimated using a multivariable logistic regression model that included baseline age, age^2^, gender, duration of diabetes, retinopathy, obesity, diabetes-related hospitalization, DPP-4 inhibitor, insulin, ACEI, BB, CCB, thiazide diuretics, other diuretics, nitrates, digoxin, other antiplatelet drugs, and warfarin and was compared to SU plus metformin in the ischemic stroke analysis (C-statistic = 0.601).

^e^ The propensity of receiving a DPP-4 inhibitor plus metformin was estimated using a multivariable logistic regression model that included baseline age, age^2^, gender, retinopathy, nephropathy, hypertension, α-glucosidase inhibitors, TZDs, insulin, ACEIs, ARBs, BBs, CCBs, thiazide diuretics, other diuretics, nitrates, digoxin, aspirin, other antiplatelet drugs, warfarin, statins, and nonsteroidal anti-inflammatory drugs and was compared to pioglitazone plus metformin in the total cardiovascular disease outcome analysis (C-statistic = 0.646).

^f^ The propensity of receiving a DPP-4 inhibitor plus metformin was estimated using a multivariable logistic regression model that included baseline age, age^2^, gender, duration of diabetes, retinopathy, neuropathy, hypertension, obesity, diabetes-related hospitalization, metformin, TZDs, α-glucosidase inhibitors, meglitinides, DPP-4 inhibitor, insulin, ACEIs, BBs, CCBs, other diuretics, nitrates, digoxin, other antiplatelet drugs, and warfarin and was compared to pioglitazone plus metformin in the MI outcome analysis (C-statistic = 0.672).

^g^ The propensity of receiving a DPP-4 inhibitor plus metformin was estimated using a multivariable logistic regression model that included baseline age, age^2^, gender, duration of diabetes, retinopathy, neuropathy, nephropathy, peripheral vascular disease, hypertension, dyslipidemia, diabetes-related hospitalization, total number of hypoglycemic agents used, Charlson score, metformin, SUs, meglitinides, DPP-4 inhibitor, insulin, ACEIs, ARBs, BBs, CCBs, other diuretics, nitrates, digoxin, other antiplatelet drugs, warfarin, and statins and was compared to pioglitazone plus metformin in the HF outcome analysis (C-statistic = 0.640).

^h^ The propensity of receiving a DPP-4 inhibitor plus metformin was estimated using a multivariable logistic regression model that included baseline age, age^2^, gender, retinopathy, nephropathy, hypertension, dyslipidemia, diabetes-related hospitalization, metformin, SUs, α-glucosidase inhibitors, DPP-4 inhibitor, insulin, ACEIs, ARBs, BBs, CCBs, thiazide diuretics, other diuretics, digoxin, aspirin, other antiplatelet drugs, and warfarin and was compared to pioglitazone plus metformin in the ischemic stroke outcome analysis (C-statistic = 0.606).

## Discussion

The results of this population-based cohort study showed that the use of a sulfonylurea derivative plus metformin was associated with increased risks of total CVD, MI, and IS compared with the use of a DPP-4 inhibitor plus metformin. In addition, we found that the use of pioglitazone plus metformin was associated with an increased risk of HF compared with a DPP-4 inhibitor plus metformin. However, lower risks for total CVD and IS were observed in patients receiving pioglitazone plus metformin.

The findings of this study were similar to those of a previous meta-analysis that examined the risk for major CVEs among patients using a DPP-4 inhibitor relative to the placebo or other OAs. In this meta-analysis, DPP-4 inhibitors were associated with a lower risk of major CVEs, although the result was not statistically significant when subgroup analysis was conducted to compare the DPP-4 inhibitor and sulfonylurea use [8]. Recently published data from RCTs including patients at a higher cardiovascular risk showed that DPP-4 inhibitor and placebo groups did not differ significantly with respect to major CVEs [11–12]; however, comparison of these data with our data were difficult due to the differences in patient characteristics and the comparison group. The present study found significantly increased risks of total CVD, MI, and IS for treatment with a sulfonylurea drug plus metformin compared to treatment with a DPP-4 inhibitor plus metformin, suggesting a reduced risk in DPP-4 inhibitor users. Indeed, some preclinical and clinical studies have demonstrated that DPP-4 inhibitors may provide beneficial effects on blood pressure, lipid metabolism, inflammatory mediators, and endothelial function, although the precise underlying mechanisms for cardiovascular benefits require further investigation [23–30].

The risk of HF was associated with pioglitazone plus metformin compared with a DPP-4 inhibitor plus metformin, and this result is consistent with the fact that pioglitazone is associated with an increased risk for HF [31]. However, our study also found that pioglitazone plus metformin was associated with lower risks of total CVD and IS than a DPP-4 inhibitor plus metformin. Although the effect of pioglitazone compared with DPP-4 inhibitors on cardiovascular outcomes has not been previously reported, a meta-analysis of 19 trials comparing pioglitazone with a placebo or other OAs found that pioglitazone was associated with a significantly lower risk of stroke and the composite of MI, stroke, or death [31]. The favorable effects of pioglitazone on stroke, death, and the composite of acute MI, stroke, HF, or death compared to rosiglitazone have also been reported in an observational study [32]. These findings suggest that the net clinical cardiovascular benefit of pioglitazone therapy could be favorable in patients without a risk of HF. However, the findings of this study must be interpreted with caution because they may be subject to biases arising from confounding variables. While differences in baseline characteristics were adjusted in our analysis, this adjustment could not completely correct for the uneven distribution of unobservable factors that may have influenced the cardiovascular risks. Moreover, channeling patients at higher risk to receive a DPP-4 inhibitor plus metformin may have occurred because this is a newer therapy with uncertain cardiovascular risk. Other authors have observed that sitagliptin users show higher proportions of comorbidities and a greater use of prescription medications and physician visits compared to users of other OAs [33–34], and these findings suggest that patients receiving a DPP-4 inhibitor may be at higher risk for clinical and health outcomes such as CVEs due to their higher baseline risk.

In the stratified analysis, the adjusted HRs for pioglitazone plus metformin compared with a DPP-4 inhibitor plus metformin were significantly reduced for total CVD and IS among males; however, the adjusted HRs were not statistically significant among females. The adjusted HR of HF for pioglitazone plus metformin was not significantly increased among males; however, the corresponding HR was statistically significantly increased among females. This result suggests a potential difference in the cardiovascular effect of pioglitazone on males and females. Indeed, pioglitazone is known to influence estrogen function and sex hormone-binding globulin expression [35–36], which may have contributed to the observed gender difference.

A study outcome occurred shortly after insulin initiation would be the effect of study therapy on cardiovascular outcomes, whereas this event was not defined as an occurrence of study outcome according to the operational definition of at-risk person time; this may bias study results. However, analysis including that event, which is an outcome of interest occurred after therapy switch, may also be subject to bias because that accounts for the different diabetes therapy conditions.

This study possesses several unique strengths. First, this study included the entire population of patients with type 2 diabetes using the national health insurance claims database; therefore, this study could show representative results in the population of approximately 50 million residing in Korea. In addition, our study population represents the patient population in real-world practice settings. Second, head‐to‐head comparisons of a DPP-4 inhibitor plus metformin with a sulfonylurea drug plus metformin and pioglitazone plus metformin were conducted. Consequently, our data provide information on the comparative effectiveness of these therapies. In addition, the combined use of DPP-4 inhibitors with metformin was covered by health insurance in Korea, whereas monotherapy with DPP-4 inhibitors was not covered. Therefore, in this study, we were able to observe most of the patients who used DPP-4 inhibitors during the study period.

Our study also had some limitations. First, the accuracy of diagnoses from claims databases is limited. However, previous studies have validated ICD-10 code-based definitions for many important diseases, including diabetes, acute MI, and ischemic stroke, which were compared with medical records reviews; these reports demonstrated positive predictive values of 72.3% to 87.2% for diabetes (E10-14), >70% for MI (I21), and 83.4% for IS (I63) [37–39]. In addition, the overall positive predictive value of the diagnosis was approximately 70% [40]. Second, clinical information, such as hemoglobin A1c levels, was not available; thus, the degree of glycemic control or the grading of diabetes severity was not well captured, which could have influenced the incidence of the outcomes. However, hospitalizations for diabetes and the total number of hypoglycemic agent classes used in the year prior to the index date were measured and adjusted for as a proxy of the degree of glycemic control. The presence of microvascular complications and peripheral vascular disease were also determined and adjusted for as a proxy of diabetes severity. Third, the maximum follow-up observation time was 2.1 years (25 months) after the index date. Even if a meta-analysis found that DPP-4 inhibitors were also associated with a significantly lower risk of major CVEs in the subgroup analysis of trials of relatively short duration (<52 weeks) [8], their long-term effects on cardiovascular outcomes remain to be established in future and ongoing studies. Fourth, although numerous potential and measurable cardiovascular risk factors were adjusted for, there is the possibility that the results were affected by unmeasured confounders, which is a universal problem with all observational studies.

In conclusion, our data suggest that, compared with a DPP-4 inhibitor plus metformin, treatment with a sulfonylurea derivative plus metformin was associated with increased risks of total CVD, MI, and ischemic stroke. Thus, adding a DPP-4 inhibitor instead of a sulfonylurea drug as second-line treatment to first-line metformin should be considered. Our results also found that the use of pioglitazone plus metformin was associated with decreased total CVD and IS risks compared with a DPP-4 inhibitor plus metformin. Although fluid retention and heart failure are more frequent with pioglitazone treatment, the beneficial effects of pioglitazone on total CVD and IS provide a well-selected treatment option for patients with type 2 diabetes and a lower risk of HF. However, this is an observational study, and there may be residual confounding variables; thus, large, ongoing long-term clinical trials to specifically address the cardiovascular risk of DPP-4 inhibitors will offer more insight.

## Supporting Information

S1 Fig Distribution of the estimated propensity of receiving dipeptidyl peptidase-4 inhibitor plus metformin versus a sulfonylurea drug plus metformin in the total cardiovascular disease outcome analysis (A), myocardial infarction (B), heart failure (C), and ischemic stroke (D). DPP-4 = dipeptidyl peptidase-4; SU = sulfonylurea.
(TIF)

S2 Fig Distribution of the estimated propensity of receiving dipeptidyl peptidase-4 inhibitor plus metformin versus a pioglitazone plus metformin in the total cardiovascular disease outcome analysis (A), myocardial infarction (B), heart failure (C), and ischemic stroke (D). DPP-4 = dipeptidyl peptidase-4.
(TIF)

S1 Table Baseline Covariate Definitions and Codes. (DOCX)
