# Supplementary figures and images for: Differential Cardiovascular Outcomes after Dipeptidyl Peptidase-4 Inhibitor, Sulfonylurea, and Pioglitazone Therapy, All in Combination with Metformin, for Type 2 Diabetes: A Population-Based Cohort Study
Source: PLoS One. 2015 May 20;10(5):e0124287. doi: 10.1371/journal.pone.0124287 (PMC4439115; doi:10.1371/journal.pone.0124287)

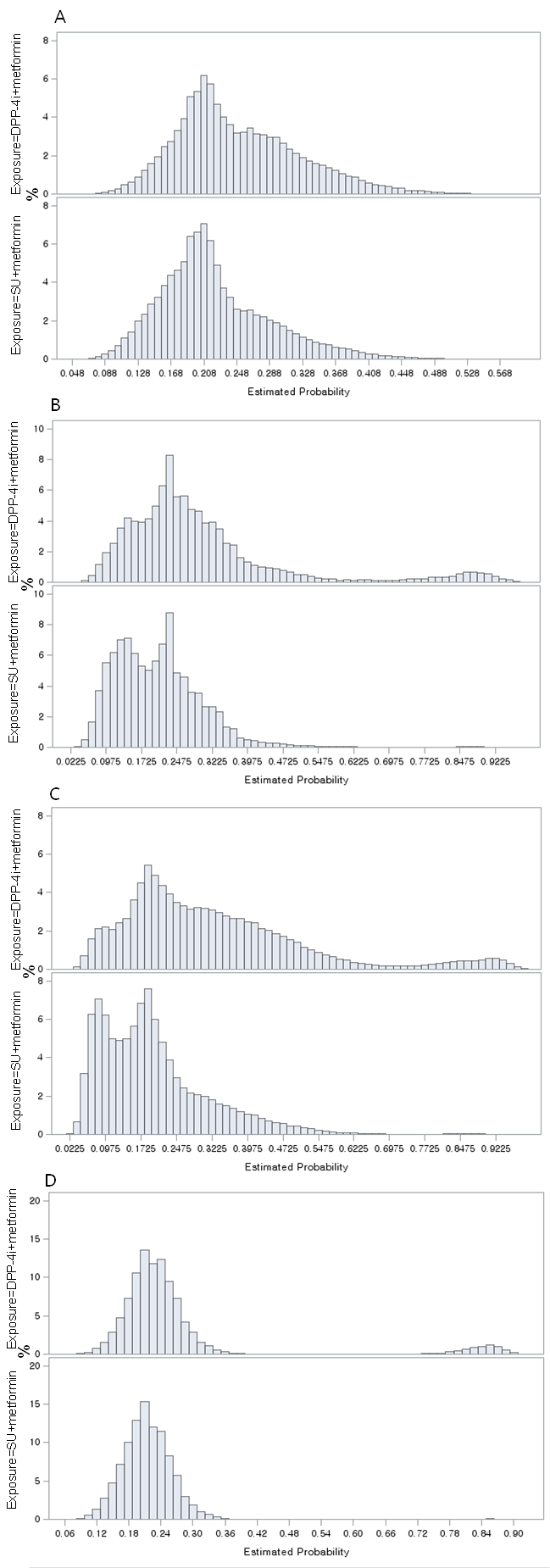

Supplement: S1 Fig — DPP-4 = dipeptidyl peptidase-4; SU = sulfonylurea. (TIF) [file pone.0124287.s001.tif]

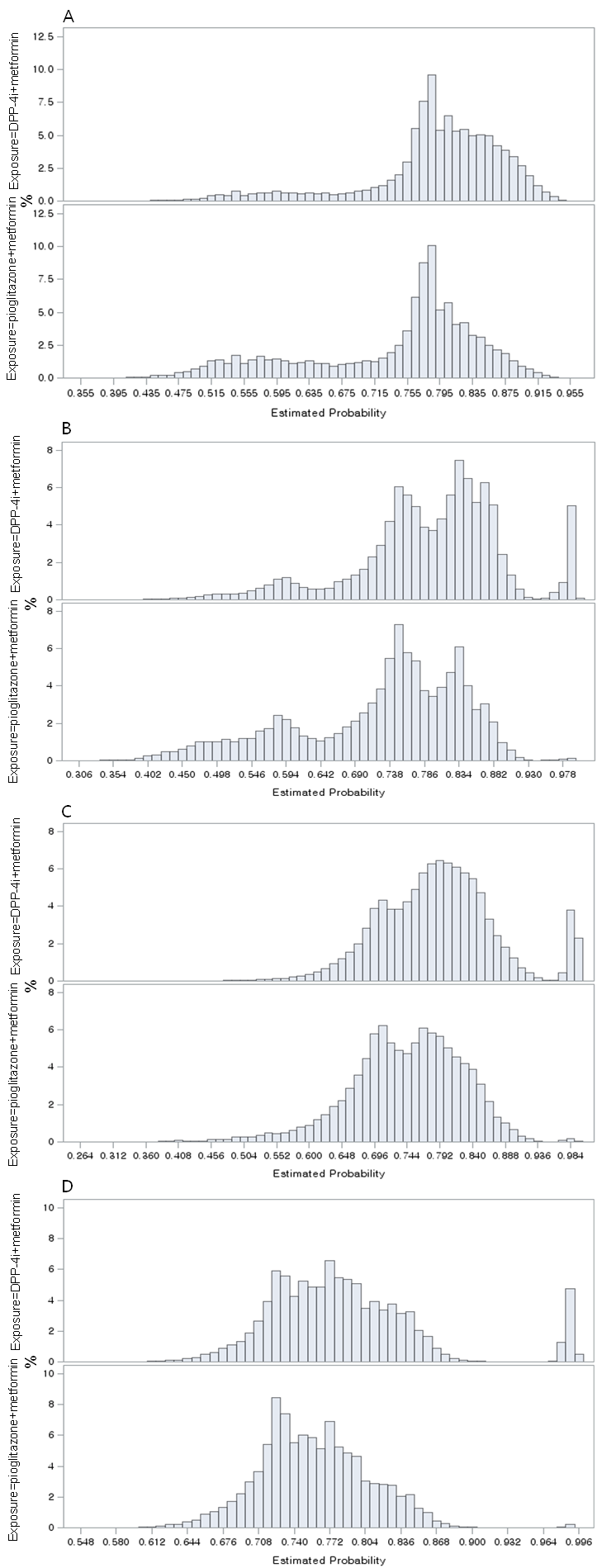

Supplement: S2 Fig — DPP-4 = dipeptidyl peptidase-4. (TIF) [file pone.0124287.s002.tif]
