# Supplementary material for: Differential Cardiovascular Outcomes after Dipeptidyl Peptidase-4 Inhibitor, Sulfonylurea, and Pioglitazone Therapy, All in Combination with Metformin, for Type 2 Diabetes: A Population-Based Cohort Study
Source: PLoS One. 2015 May 20;10(5):e0124287. doi: 10.1371/journal.pone.0124287 (PMC4439115; doi:10.1371/journal.pone.0124287)
Supplement: S1 Table — (DOCX) [file pone.0124287.s003.docx]

**SUPPLEMENTAL MATERIAL**

Supplemental Table 1. Baseline Covariate Definitions and Codes

| Requires 3 outpatient or 1 inpatient diagnosis or procedure code in the 1 year prior to the index date. | |
| --- | --- |
| Microvascular complications of diabetes |  |
| Retinopathy | ICD-10 codes E10.3, E11.3, E12.3, E13.3, E14.3, H36.0, H33, H54, H34, and H35 |
| Neuropathy | ICD-10 codes E10.5, E11.5, E12.5, E13.5, E14.5, G56, G57, G58, G59, G64, G62.9, G63.2, G90.0, G90.8, G90.9, G99.0, G99.1, G53.8 |
| Nephropathy | ICD-10 codes E10.2, E11.2, E12.2, E13.2, E14.2, N03, N05, N17, N18, N19, N28.9, N25, Z49, Z99.2, Z94.0  Procedure code R3280 (renal transplantation) |
| Other comorbidities |  |
| Peripheral vascular disease | ICD-10 codes E10.5, E11.5, E12.5, E13.5, E14.5, I73.8, I73.9, I77.1, I79.0*, I79.2*, I798, K55.1, K55.8, K55.9, Z95.8, Z95.9, L97, R02, S807, S808, S809, S817, S818, S819, S907, S908, S909, S91.x, T13.x, Z894  Procedure codes N0571 (Amputation of Extremities, Pelvis), N0572 (Amputation of Extremities, Thigh), N0573 (Amputation of Extremities, Upper Arm, Forearm, Lower Leg), N0574 (Amputation of Extremities, Hand, Foot), N0575 (Amputation of Extremities, Finger, Toe) |
| Hypertension | ICD-10 codes I10, I11, I12, I13, I15 |
| Dyslipidemia | ICD-10 code E78 |
